# The impact of clothing style on bone mineral density among post menopausal women in Morocco: a case-control study

**DOI:** 10.1186/1471-2458-6-135

**Published:** 2006-05-19

**Authors:** Fadoua Allali, Siham El Aichaoui, Bouchra Saoud, Houda Maaroufi, Redouane Abouqal, Najia Hajjaj-Hassouni1

**Affiliations:** 1 Department of Rheumatology, El Ayachi University-Hospital, Sale, Morocco; 2 Laboratory of Biostatistical, Clinical Research and Epidemiology (LBRCE). Faculty of Medicine and Pharmacy, Rabat, Morocco

## Abstract

**Background:**

The clothing style is an important factor that influences vitamin D production and thus bone mineral density. We performed a case-control study in order to evaluate the effect of veil wearing (concealing clothing) on bone mineral density in Moroccan post menopausal women.

**Methods:**

The cases were osteoporotic women whose disease was assessed by bone mineral density measurement. Each patient was matched with a non osteoporotic woman for age, and body mass index. All our patients were without secondary causes or medications that might affect bone density. The veil was defined as a concealing clothing which covered most of the body including the arms, the legs and the head. This definition is this of the usual Moroccan traditional clothing style.

**Results:**

178 post menopausal osteoporotic patients and 178 controls were studied. The mean age of the cases and the controls was 63.2 years (SD 7) and the mean body mass index was 32.1 (SD 8). The results of crude Odds Ratios analyses indicated that wearing a veil was associated with a high risk of osteoporosis: OR 2.29 (95% CI, 1.38–3.82). Multiparity or a history of familial peripheral osteoporotic fractures had also a significant effect on increasing the osteoporosis risk (ORs: 1.87 (95% CI, 1.05–3.49) and 2.01 (95% CI, 1.20–3.38)). After a multiple regression analysis, wearing the veil and a history of familial osteoporotic fractures remained the both independent factors that increased the osteoporosis risk (ORs: 2.20 (95% CI, 1.22–3.9) and 2.19 (95% CI, 1.12–4.29) respectively).

**Conclusion:**

our study suggested that in Moroccan post menopausal women, wearing a traditional concealing clothing covering arms, legs and head increased the risk of osteoporosis. Further studies are required to evaluate the clinical impact of the above findings and to clarify the status of vitamin D among veiled women in Morocco.

## Background

Osteoporosis is a common disorder in the elderly population, and represents one of the most significant public health problems in the world. Osteoporosis can be assessed indirectly through a non-invasive measurement of bone mineral density (BMD). BMD is influenced by both genetic and environmental factors, and possibly interactions between them [1]. Vitamin D insufficiency (levels between 5 and 20 ng/ml) leads to calcium malabsorption, secondary hyperparathyroidism and decreased BMD [2,3] while severe hypovitaminosis D (levels below 5 ng/ml) leads to osteomalacia. Vitamin D is synthesized in the skin after sunlight exposure or can be obtained through a balanced dietary intake. However, because a few foods provide a natural source of vitamin D, and because supplementation of food is often unreliable, skin synthesis is thought to constitute the major source [4]. Hence, a limited skin exposure to sunlight has been presumed to be the major cause of Vitamin D-deficiency. Thus, in adults living in the Northern United States and Europe, there is not only a higher prevalence of hypovitaminosis D than in adults living in regions neighboring the equator, but also a large seasonal variation, with higher levels at the end of summer and lower levels at the end of winter [5-8]. Regarding this significant role of sunlight in vitamin D synthesis, it would be quite logical to suggest a low prevalence of vitamin D deficiency in tropical countries. However, the studies carried out in some of these countries, such as Turkey, India, Iran and Saudi Arabia during the preceeding two decades have shown a high prevalence of vitamin D deficiency [9-12]. UV-B radiation depends on many factors for instance latitude, season and day time of sunlight exposure. Moreover, the EPIDOS Study Group showed that vitamin D status of an elderly and healthy population living at home depends mainly on lifestyle [13]. The clothing style is an important lifestyle factor that influences vitamin D production [14-18]. Indeed, it has been shown that veiled women had a lower 25 OH vitamin D status [19,20] and a higher bone turnover than unveiled women [21,22].

In Morocco, most of the women even unveiled wear a scarf and a Djellaba which is a long, loose-fitting, and hooded garment with long sleeves that is worn over casual clothes. Thus, their sunlight exposure might be insufficient.

We performed this study in order to evaluate the effect of the Moroccan concealing clothing style on BMD in post menopausal women.

## Methods

### Study design

It was a case-control study. Its objective was to evaluate the impact of the clothing style on BMD among post menopausal women leaving in Morocco. The Medical research ethics comity of the faculty of medicine of Rabat approved the study, and all participants provided written consent.

### Cases and controls

The cases were volunteer osteoporotic women. Osteoporosis was assessed by BMD and defined according to the World Health Organisation (WHO) (< – 2,5 standard deviation of normal values for young people). Each patient was matched with a volunteer non osteoporotic woman for age, and body mass index.

Eligible patients and controls were post menopausal women living in urban centers of Morocco who were referred to our outpatient Bone Densitometry Center from february 2004 to march 2005. All subjects were referred to this center for assessment of osteoporosis risk factors, including menopause.

Study inclusion criteria were: (1) postmenopausal status (at least 1 year of menopause). Exclusion criteria included having a history of: (1) taking drugs known to influence bone metabolism in the past 2 years, such as vitamin D, calcium, corticosteroids, bisphosphonates and hormone replacement therapy; (2) musculoskeletal, thyroid, parathyroid, adrenal, hepatic, or renal disease; (3) malignancy; and (4) hysterectomy.

### Data collection and measurements

Each patient completed a questionnaire on sociodemographic parameters and osteoporosis risk factors. Information regarding veil was recorded. The definition of veil was that of a concealing clothing, i.e. a clothing style not only limited to the Islamic dressing habits but also including the Moroccan traditional clothing which covers most of the body including the arms, the legs and the head.

The age of menopause, the time since menopause, the personal history of cigarette smoking or alcohol intake and the number of pregnancies were recorded. We also collected data related to the personal and familial history of peripheral osteoporosis fractures (including proximal femoral fractures).

*Anthropometric data*: weight and height were measured without clothes nor shoes at the time of bone densitometry measurements. The body mass index (BMI) was calculated as body weight (kg)/height (m^2^).

*Bone mineral density *(*BMD*) of lumbar spine and total hip (bilateral proximal femur) was measured by dual-energy X-ray absorptiometry with a Lunar prodigy densitometer. Both *T *and *Z *scores were obtained. In the *T*-score calculations, the manufacturer's ranges for European reference population were used because of the absence of a Moroccan data base.

### Statistical analysis

Results are expressed as mean and standard deviation. We conducted conditional logistic regression analyses using SPSS. Risk estimation is presented as odds Ratios (ORs) with 95% confidence intervals (CIs). *P *values are 2 sided and were considered statistically significant if less than 0.05.

Odds Ratios were adjusted for the potential confounders age, body mass index (BMI; <25, 25–29.9, ≥ 30), time since menopause (< 5 years, 5–10 years, >10 years), personal and familial history of peripheral osteoporotic fractures, number of pregnancies as well as smoking status (yes, none).

## Results

We studied 178 post menopausal osteoporotic patients and 178 controls matched by age and body mass index. The characteristics of the cases and the controls are summarized in table 1. The mean age of the cases and the controls was 63.2 years (SD 7) and the mean body mass index was 32.1 (SD 8). 83.7% of the osteoporotic patients were veiled vs 69.1% among non osteoporotic patients (*p *< 0.001).

The crude and adjusted ORs for osteoporosis risk factors are shown in table 2. The results of crude analyses indicated that wearing the veil was associated with a high risk of osteoporosis: OR 2.29 (95% CI, 1.38–3.82; *P *< 0.001). Multiparity (more than 5 children) or a history of familial peripheral osteoporotic fractures had also a significant effect on increasing osteoporosis risk: ORs 1.87 (95% CI, 1.05–3.49; *P *< 0.005); and 2.01 (95% CI, 1.20–3.38;*P *< 0.05) whereas time since menopause, personal history of fractures, cigarette smoking and alcohol intake were not associated with osteoporosis risk.

After a multiple regression analysis, wearing the veil and a history of familial osteoporotic fractures remained the both independent factors for increasing osteoporosis risk (ORs: 2.20 (95% CI, 1.22–3.9; *P *< 0.005) and 2.19 (95% CI, 1.12–4.29; *P *< 0.05) respectively).

## Discussion

Our study showed that a concealing clothing style covering arms, legs and head increased the risk of osteoporosis by 2.2. The cases and the controls had the same origin and lived in the same country. There was no statistical difference in terms of age, body mass index, cigarette smoking and alcohol intake. All our patients were without secondary causes or medications that might affect bone density.

After controlling for other osteoporosis risk factors such as time since menopause, number of pregnancies, smoking, personal and familial history of peripheral osteoporotic fractures, osteoporosis risk was found to be significantly associated with veil wearing. The lack of relationship between a personal history of fractures and BMD might be explained by the low percentage of personal fractures in those patients.

Our study did not assess daily dietary vitamin D intake. However, it is well known that natural nutrients are not sufficient sources of vitamin D to supply the body requirements. Therefore, supplementation is often needed. When not afforded, which is the case in Morocco, the main production of vitamin D is provided by UV sunlight.

Sunlight is abundant in Morocco throughout the year. However the extent of sunlight-UV exposure is determined much more by lifestyle (which includes clothing style) rather than outdoor UV irradiance.

The influence of clothing style on vitamin D status has been the subject of a few studies. Two reports from Saudi Arabia [23] and Kuwait [19] revealed vitamin D deficiency among people from the Arabian Gulf. This deficiency was more found among veiled Kuwaiti women than in non veiled women [19].

Diamond et al [16] showed that wearing a veil was an independent predictor factor of hypovitaminosis D. Rassouli [11] et al showed a 25 OH vitamin D deficiency in 36 % of Iranian women wearing a garb. This deficiency was correlated with spine BMD but not with femoral BMD.

The interactive effect of concealing clothes on BMD had, to our knowledge, never been studied in African women whereas some studies have been carried out on the vitamin D status. For instance, in Tunisia, Meddeb [24] et al found a vitamin D deficiency in 47.6% of the studied female population. Factors associated with D hypovitaminosis were multiparity, menopause, wearing the veil, calcium and vitamin D intake. After logistic regression only multiparity and vitamin D dietary intake remained as independent predictive factors of vitamin D deficiency. Bone mineral density wasn't performed in this study.

The results of our study may be added to those of other countries the vestimentary habits of which are almost similar to ours. It is also important to clarify the status of vitamin D and the causes of the observed deficiencies in order to provide preventive measures. It is also obviously necessary to study the correlations between the vitamin D status and BMD and therefore the risk of osteoporotic fractures among veiled women in theses countries.

## Conclusion

Our study suggested that in Moroccan post menopausal women, wearing traditional clothes, covering arms, legs and head, increased the risk of osteoporosis. Urgent measures are needed in our country to prevent long term complications related to bone mineral density decrease and therefore related to the risk of fractures. These measures should include the assessment of vitamin D status among the population and perhaps more particularly in women who adopt concealing clothing, decreasing, among other factors, cutaneous surface available for a sufficient solar exposure. It may be also important to fortify milk, dairy products, or other food and beverages in order to assure an adequate vitamin D intake.

Until these general measures become effective, more research is needed to evaluate the clinical impact of the above findings.

## Competing interests

The author(s) declare that they have no competing interests.

## Authors' contributions

N. Hajjaj-Hassouni and F. Allali conceived the study and supervised its design, execution, and analysis and participated in the drafting and critical review of the manuscript. R. Abouqal participated in the concept and the design of the study, did data management and statistical analyses and participated in the drafting and critical review of the manuscript. All the other authors enrolled patients, participated in data acquisition and critical revision of the manuscript. F. Allali wrote the paper, all the authors read and approved the final manuscript.

## Pre-publication history

The pre-publication history for this paper can be accessed here:



## Figures and Tables

**Table 1:** Characteristics of case patients with osteoporosis and matched controls

	**Cases (n = 178)** **Mean ± SD**	**Controls (n = 178)** **Mean ± SD**
Age (years)	63,2 ± 7,4	62,6 ± 7,1
Menopause age (years)	49,5 ± 5,6	50 ± 5,1
Body mass index (kg/m^2^)	32,1 ± 8,9	32 ± 8,4
Number of pregnancies	4,7 ± 2,5	4,3 ± 2,5
Lumbar BMD (g/cm^2^)	0,80 ± 0,08	1,03 ± 0,1
Lumbar T-score	-3,13 ± 0,6	-1,1 ± 0,9
Total hip BMD (g/cm^2^)	0,7 ± 0,1	0,9 ± 0,1
Total hip T-score	-1,7 ± 0,9	-0,7 ± 0,1

**Table 2:** The crude and adjusted Odds ratio for osteoporosis risk factors in case patients and matched controls

***Characteristics***	**Cases (n = 178)**	**Controls (n = 178)**	**Crude Odds Ratio (95%CI)**	***P *value**	Adjusted Odds Ratio (95%CI)*	***P *value**
**Age (years)**^†^						

< 55	30 (16.8)	28 (15.7)				
56–60	33 (18.5)	38 (21.3)				
61–65	51 (28.7)	57 (32)				
66–70	36 (20.2)	29 (16.3)				
>70	28 (15.7)	26 (14.6)				
**Body mass index (kg/m**^**2**^**)**^†^						

<25	35 (19.7)	32 (18)				
25–29.9	55 (30.9)	62 (34.8)				
≥30	88 (49.4)	84 (47.2)				
**Veil Wearing**						

No	29 (16.3)	55 (30.9)	1		1	
yes	149 (83.7)	123 (69.1)	2.29 (1.38–3.82)	0.001	2.20 (1.22–3.9)	0.008
**Time since menopause (years)**						

<5	27 (15.2)	35 (19.7)	1		1	
5–10	32 (18)	34 (19.1)	1.22 (0.6–2.4)	0.57	1.10 (0.51–2.39)	0.7
>10	119 (66.9)	109 (61.2)	1.41 (0.8–2.4)	0.22	1.08 (0.52–2.24)	0.8
**History of personal peripheral osteoporotic fractures**						

No	139 (78.1)	149 (83.7)	1		1	
yes	39 (21.9)	29 (16.3)	1.44 (0.84–2.45)	0.17	1.34 (0.75–2.38)	0.3
**History of familial peripheral osteoporotic fractures**						

No	147 (82.6)	160 (89.9)				
yes	31 (17.4)	18 (10.1)	1.87 (1.05–3.49)	0.04	2.19 (1.12–4.29)	0.02
**Number of pregnancies**						

<3	43 (31.4)	39 (27.7)	1		1	
3–5	67 (48.9)	89 (63.1)	0.68 (0.3–1.1)	0.16	0.65 (0.37–1.1)	0.1
> 5	27 (19.7)	13 (9.2)	2.01 (1.20–3.38)	0.007	1.34 (0.75–2.38)	0.3
**Cigarette smoking**						

No	175 (98.3)	171 (96.1)	1		1	
yes	3	7	0.42 (0.1–1.6)		0.66 (0.14–2.9)	0.5
**Alcohol intake**						

No	176 (97.7)	177 (99.4)	1			
yes	2 (1.13)	1 (0.6)	1 (0.06–16)			

Abbreviation: CI, confidence interval

* Adjusted for the other categorical parameters in this table except for alcohol intake.

† Matching variables.
